# CAN NUDGES REDUCE THE SHARING OF MISINFORMATION ONLINE? EXPERIMENTAL EVIDENCE ACROSS THE LIFESPAN

**DOI:** 10.1093/geroni/igae098.4137

**Published:** 2024-12-31

**Authors:** Ryan Moore, Li Chu, Jeffrey Hancock, Laura Carstensen

**Affiliations:** Stanford University, Stanford, California, United States; Stanford University, Stanford, California, United States; Stanford University, Stanford, California, United States; Stanford University, Stanford University, California, United States

## Abstract

Observational data on online behavior indicates that older adults are more likely to share misinformation than younger adults. Thus, identifying scalable strategies to reduce older adults’ sharing of misinformation is imperative. This study investigates the potential for 1-sentence text-based nudges appearing alongside online news to reduce intentions to share misinformation. A lifespan sample of American adults (aged 18-99, M=49.1, SD=20.8) was shown online news headlines fact-checked as true or false by professional fact-checkers and asked about their intentions to share each headline. Alongside each headline, participants saw nudges that varied in two ways: (1) whether they were emotionally meaningful or not and (2) whether they were presented in a novel manner or not. Drawing on socioemotional selectivity theory, which posits that as people’s time horizons shrink they prioritize emotional meaning, we expected that emotionally meaningful nudges would be more likely to reduce older adults’ sharing of misinformation. Interestingly, in contrast to studies based on online behavioral data, we found that in a survey context older adults were less likely to report intentions to share misinformation online than younger adults, even after controlling for baseline sharing tendencies and other covariates. We found that novel nudges were more effective than non-novel nudges at reducing intentions to share misinformation across all ages, and that novel nudges were more effective than accuracy nudges, another popular intervention (Pennycook et al. 2021). Emotionally meaningful nudges were not more effective for older adults. This study draws implications for designing effective interventions against misinformation sharing across the lifespan.

